# Reagent-free detection of *Plasmodium falciparum* malaria infections in field-collected mosquitoes using mid-infrared spectroscopy and machine learning

**DOI:** 10.1038/s41598-024-63082-z

**Published:** 2024-05-27

**Authors:** Emmanuel P. Mwanga, Prisca A. Kweyamba, Doreen J. Siria, Issa H. Mshani, Idrisa S. Mchola, Faraja E. Makala, Godian Seleman, Said Abbasi, Sophia H. Mwinyi, Mario González-Jiménez, Klaas Waynne, Francesco Baldini, Simon A. Babayan, Fredros O. Okumu

**Affiliations:** 1 Environmental Health and Ecological Sciences Department, Ifakara Health Institute, Morogoro, Tanzania; 2 School of Biodiversity, One Health and Veterinary Medicine, University of Glasgow, Glasgow, G12 8QQ UK; 3 Swiss Tropical and Public Health Institute, Kreuzstrasse 2, 4123 Allschwil, Switzerland; 4 University of Basel, Petersplatz 1, 4001 Basel, Switzerland; 5 School of Chemistry, The University of Glasgow, Glasgow, G12 8QQ UK; 6 School of Public Health, Faculty of Health Sciences, University of the Witwatersrand, Johannesburg, South Africa; 7 School of Life Science and Bioengineering, The Nelson Mandela African Institution of Science and Technology, P. O. Box 447, Arusha, Tanzania

**Keywords:** Machine learning, Infrared spectroscopy, Malaria, Entomology

## Abstract

Field-derived metrics are critical for effective control of malaria, particularly in sub-Saharan Africa where the disease kills over half a million people yearly. One key metric is entomological inoculation rate, a direct measure of transmission intensities, computed as a product of human biting rates and prevalence of *Plasmodium* sporozoites in mosquitoes. Unfortunately, current methods for identifying infectious mosquitoes are laborious, time-consuming, and may require expensive reagents that are not always readily available. Here, we demonstrate the first field-application of mid-infrared spectroscopy and machine learning (MIRS-ML) to swiftly and accurately detect *Plasmodium falciparum* sporozoites in wild-caught *Anopheles funestus*, a major Afro-tropical malaria vector, without requiring any laboratory reagents. We collected 7178 female *An. funestus* from rural Tanzanian households using CDC-light traps, then desiccated and scanned their heads and thoraces using an FT-IR spectrometer. The sporozoite infections were confirmed using enzyme-linked immunosorbent assay (ELISA) and polymerase chain reaction (PCR), to establish references for training supervised algorithms. The XGBoost model was used to detect sporozoite-infectious specimen, accurately predicting ELISA and PCR outcomes with 92% and 93% accuracies respectively. These findings suggest that MIRS-ML can rapidly detect *P. falciparum* in field-collected mosquitoes, with potential for enhancing surveillance in malaria-endemic regions. The technique is both fast, scanning 60–100 mosquitoes per hour, and cost-efficient, requiring no biochemical reactions and therefore no reagents. Given its previously proven capability in monitoring key entomological indicators like mosquito age, human blood index, and identities of vector species, we conclude that MIRS-ML could constitute a low-cost multi-functional toolkit for monitoring malaria risk and evaluating interventions.

## Introduction

Vector surveillance is an essential component of malaria control and elimination, and generally includes an assessment of prevailing transmission intensities, the behaviours of different vector species and the responsiveness of these species to different interventions^1^. The most direct metric of malaria transmission intensities is the entomological inoculation rate (EIR), which is the number of infectious bites per person in a unit time, and is defined as the product of the human biting rate (HBR) and proportion of the biting mosquitoes that have *Plasmodium* sporozoite in their salivary glands^2,3^. While other entomological parameters such as mosquito abundance, age structure, daily survival probabilities, larval densities and blood-feeding preferences are important, EIR is also used to estimate the level of exposure and analyse the effectiveness of control programs. However, current reports indicate that not all endemic countries possess transmission intensity data or measure sporozoite rates^4,5^. Arguably, therefore, having a simpler method for testing samples might improve these surveillance capabilities in endemic countries.

*Plasmodium* infections in mosquitoes can be detected using various techniques, the main ones being enzyme-linked immunosorbent assay (ELISA) and polymerase chain reaction (PCR), which are both used widely, especially in research settings^6–10^. Other more traditional approaches include dissection and microscopic examination of the salivary glands^8^ and Loop-Mediated Isothermal Amplification (LAMP) assays^11^. These techniques, despite being key features in many laboratories, present several challenges, which often limit their adoption for programmatic use beyond research projects. For example false positivity rates have been reported in ELISA assays, especially where malaria vector species with zoonotic behaviours are screened, in which cases a number of non-target protozoans may be picked up in the assays, potentially leading to an overestimation of EIR^12,13^. More importantly, despite their benefits attained by both PCR and ELISA, PCR is generally expensive due to the cost of reagents. Moreover, the reagents for both PCR and ELISA are often not readily available in the localities where they are most needed. They are also time-consuming, requiring significant efforts and specialized laboratory facilities for sample preparation and processing^14,15^. Lastly, all the methods, including hand dissections of the salivary glands require highly trained and experienced personnel. These challenges underscore the critical necessity for innovative approaches that not only achieve high accuracy in detecting malaria parasites in mosquitoes but are also cost-effective, rapid, and user-friendly. Such a system would be beneficial in low-income, malaria-endemic countries, where the WHO's recommendation to incorporate surveillance as a fundamental pillar of malaria programs^1^ is hindered by the absence of easily scalable systems for effective surveillance.

Recently, the use of infrared spectroscopy, specifically near-infrared spectroscopy (NIRS, 12,500–4000 cm^−1^ frequencies of the electromagnetic spectrum), has shown potential for detecting the presence of *Plasmodium spp.* in *Anopheles* mosquitoes under controlled laboratory settings^15,16^. However, in a field validation of this technique, the predictive models could not distinguish between sporozoite-infectious and non-infectious mosquitoes^17^. Mid-infrared spectroscopy (MIRS), which uses frequencies between 4000 and 400 cm^−1^, can provide clearer peaks with more detailed information than NIRS^18,19^, and has been hypothesized to carry greater potential for such applications. Advancements in machine learning and deep learning algorithms are enhancing the potential of spectroscopic data analysis by enabling more detailed examination. This advancement allows for better specimen classification and a more detailed understanding of how samples differ in their biochemical composition^20–25^.

By integrating MIRS spectroscopic techniques and machine learning approaches, it has been possible to measure multiple entomological and parasitological indicators of malaria transmission. Examples include identifying epidemiologically relevant species and age groups of *Anopheles* mosquitoes^22,24,25^, evaluating the blood-feeding histories of mosquitoes to determine preferences for either humans or other vertebrates^21^, and detecting *Plasmodium falciparum* infections in human blood samples collected from malaria endemic villages^20,23,26,27^. However, the ability of MIRS to detect natural *Plasmodium* infections in wild-caught malaria vectors has not been demonstrated, a capability which is greatly needed to estimate malaria transmission intensities in endemic settings.

This current study was therefore designed to demonstrate the first field application of mid-infrared spectroscopy combined with machine learning (MIRS-ML) for rapid and accurate detection of *P. falciparum* in field-collected *An. funestus*. To achieve this, we evaluated the technique using PCR and ELISA as the ‘ground truth’ to detect *P. falciparum* sporozoites in wild-caught *Anopheles funestus,* the leading malaria vector in Tanzania^28–30^.

## Results

### Prevalence of *P. falciparum* sporozoites in *An. funestus* as detected by ELISA and PCR

The ELISA screening detected 184 positives out of the 4281 tested samples (4%) while the PCR screening method detected 144 positives out of the 2897 tested samples (5%).

### Machine learning classifications of mid infrared spectra of infectious and non-infectious *An. funestus*

**Figure 1:**
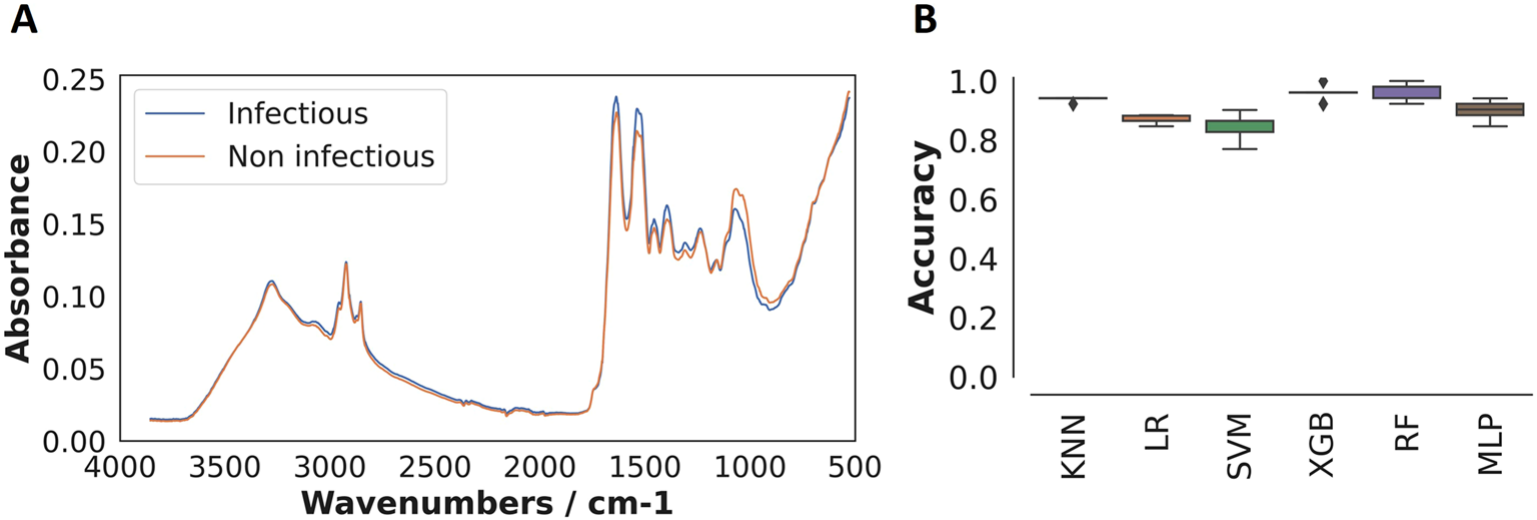
Mid-infrared spectra and machine learning analysis for classifying *An. funestus* mosquitoes based on infectious status. (**A)** Averaged mid-infrared spectra for infectious and non-infectious mosquitoes, which when analysed by the different machine learning algorithms, can enable categorization of the mosquitoes based on their infectious status. (**B)** Accuracy of standard machine learning algorithms; K-Nearest Neighbours (KNN), Logistic regression (LR), Support Vector Machine (SVM), Extreme Gradient Boosting (XGB), Random Forest (RF), and multilayer perception (MLP) in distinguishing between infectious and non-infectious mosquitoes.

To differentiate between infectious and non-infectious *An. funestus* mosquito spectra (refer to Fig. 1A), four of the six machine learning models we tested achieved prediction accuracies above 85% (Fig. 1B). Prediction accuracy refers to the proportion of correct predictions (both true positives and true negatives) made by a model out of total predictions. XGBoost was selected for further tuning of the model settings with the aim of finding the optimal combination of parameters for improved performance. This choice was made due to the capability of the XGBoost model to capture relationships between variables in the data, particularly those that do not follow straight line or a simple curve^31^.

**Figure 2:**
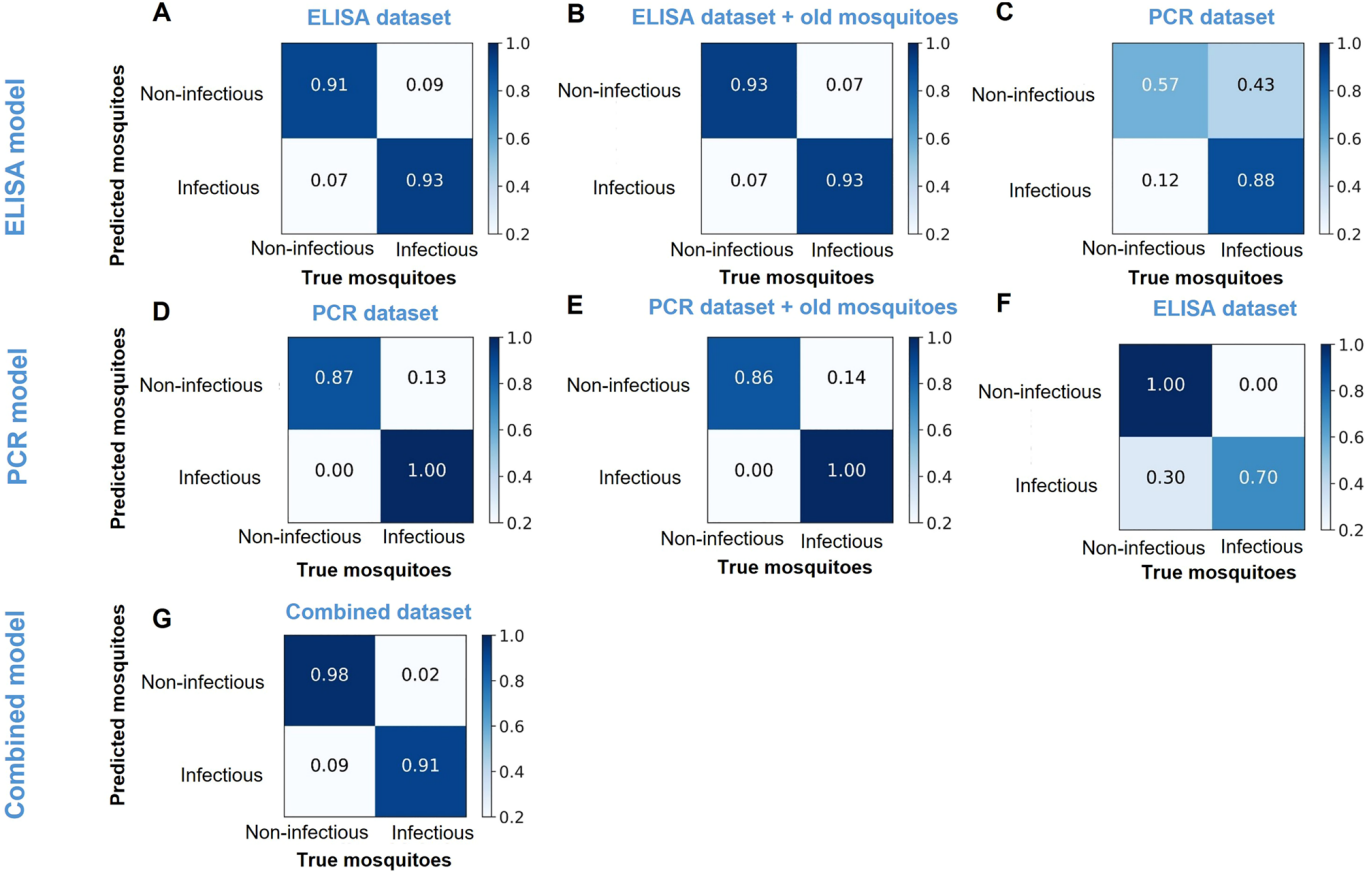
Illustrates the confusion matrices generated by the XGBoost model trained on ELISA and PCR infection datasets for predicting sporozoite infection in *An. funestus*. (**A)** Shows prediction results on an unseen segment of the ELISA dataset. (**B)** Displays predictions on augmented ELISA unseen dataset, including lab-reared 14-days old non-infectious mosquitoes. (**C)** Presents predictions on PCR dataset using the model trained on ELISA infection dataset. (**D)** Demonstrates predictions on the unseen segment of the PCR test dataset. (**E)** Shows predictions on a modified test dataset that integrates the PCR unseen test dataset with lab-reared 14-days old non-infectious mosquitoes data in the negative class. (**F)** Displays predictions on unseen ELISA test dataset using the model trained on PCR infection dataset. (**G)** Demonstrates the predictions from the model trained on the combined ELISA and PCR infection dataset for predicting sporozoite infection in *An. funestus*.

Our first XGBoost model, trained using the ELISA dataset, was able to predict the results of the ELISA test dataset with an overall accuracy of 92%. It classified spectra from the infectious and non-infectious mosquito samples with accuracies of 93% and 91% respectively (Fig. 2A). The same model was further tested to determine if mosquito age affected the classification, by introducing spectra from the known uninfected lab-reared 14-days old *An. funestus* from the laboratory (Table 2). The results showed that the performance was unaffected and was the same for classifying the new ELISA test dataset, suggesting that mosquito age did not confound the infection status in this model (Fig. 2B). The XGBoost model trained on ELISA data was also used to predict the infection labels of the spectra from mosquitoes screened for *Plasmodium* infection using PCR (Table 2). Here, the overall classification accuracy achieved by the model was 73% (Fig. 2C), though the model misclassified 43% of *Plasmodium*-negative samples (Fig. 2C); indicating limited generalizability of the model trained with ELISA derived data.

**Figure 3:**
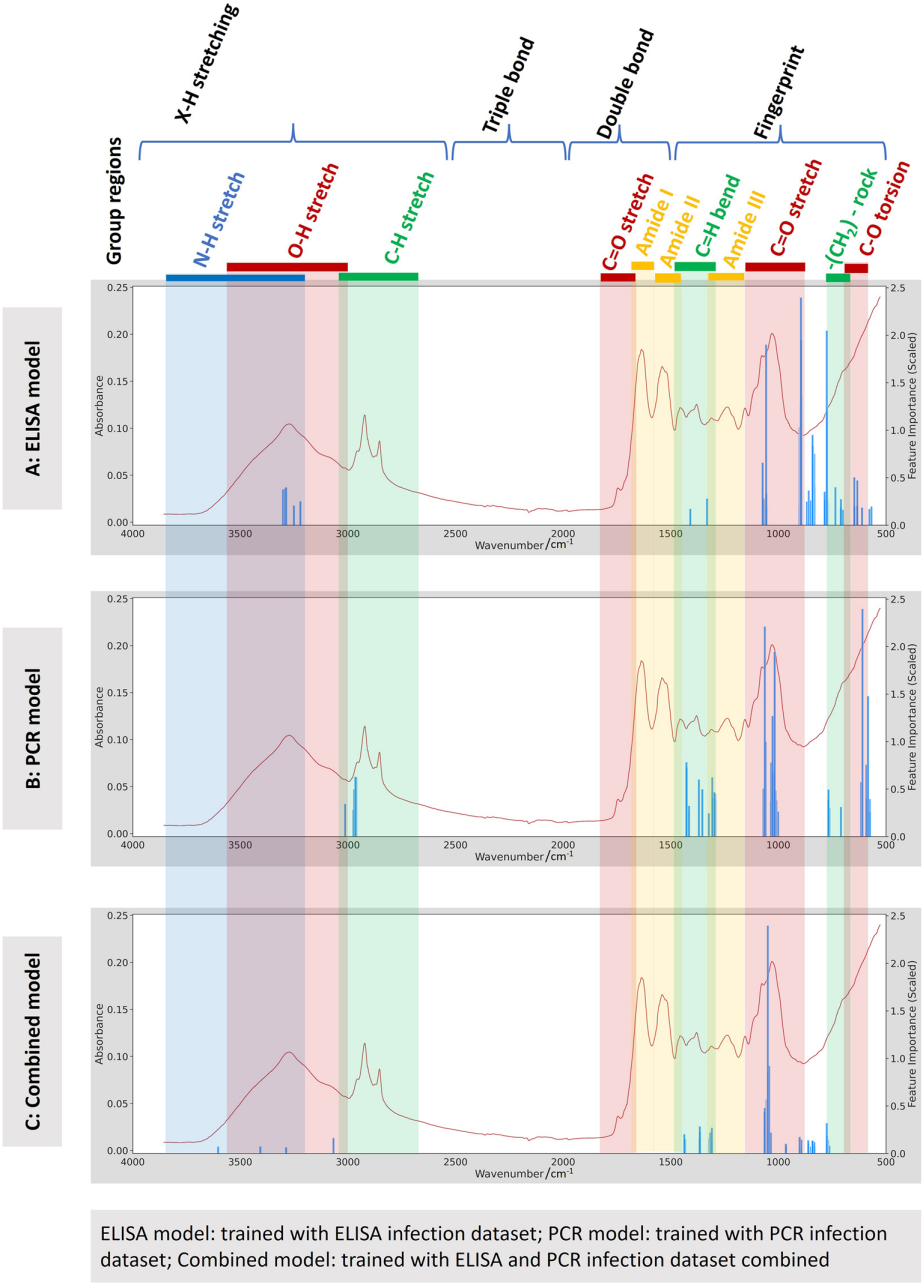
Illustrates the feature importance of the XGBoost model. The blue bars highlight the most important features for predictions, represented by scores assigned to each feature (wavenumber). The coloured stripes indicate the regions associated with different biochemical properties across the spectra. While the individual features may not be important on their own, their integration in the XGBoost Model enable the distinction of mosquitoes as either infectious or non-infectious.

To understand the biochemical signature associated to this XGBoost model, we analysed the relative importance of specific spectral features highlighted by the model. We found that the X–H region of the MIR spectra (fundamental vibrations generally due to O–H, C-H, and N–H stretches) and fingerprint region (1500–500 cm^−1^ frequencies) contributed most to the predictions (Fig. 3A).

Our second XGBoost model, trained using the PCR dataset achieved an overall classification accuracy of 94% on the PCR test dataset, predicting infectious and non-infectious mosquito samples with 87% and 100% accuracies respectively (Fig. 2D). As above, to test the influence of mosquito age on the prediction, we incorporated some old non-infectious mosquitoes (i.e. age ≥ 14 days old) into a negative class to modify the PCR test dataset and found that the classification accuracy for this augmented test dataset was identical to the model trained without the augmentation (Fig. 2E). Finally, we tested this PCR-trained model for classifying the infectious and non-infectious samples in the ELISA-derived dataset, and found an 85% classification accuracy, with the model predicting infectious and non-infectious classes at 100% and 70% accuracies respectively (Fig. 2F). The results suggest that the model, compared to the ELISA-trained model, was more effective in differentiating between *Plasmodium*-negative and positive mosquitoes. This indicates its potential as a versatile tool for analysing samples screened with various molecular techniques, including ELISA (see Fig. 2F). The analysis of important spectral features in this model showed that the spectral wavenumbers from ~ 2000 to ~ 700 cm^−1^ frequencies, which contain a complex series of absorptions, played a significant role in the predictions made by the XGBoost model (Fig. 3B).

To enhance generalizability, a new XGBoost model was trained using a combined ELISA and PCR dataset. This resulted in a prediction accuracy of 95% for the test data, including 98% accuracy for non-infectious mosquitoes and 91% for infectious ones (Fig. 2G). Notably, the crucial features contributing to this prediction, particularly from the X–H (encompassing O–H, C–H, and N–H stretching) and fingerprint regions, were also the key factors influencing the model predictions in the independent PCR and ELISA dataset trainings (Fig. 3C).

### Estimation of the EIR from the balanced test sets of PCR and ELISA infection datasets

**Figure 4:**
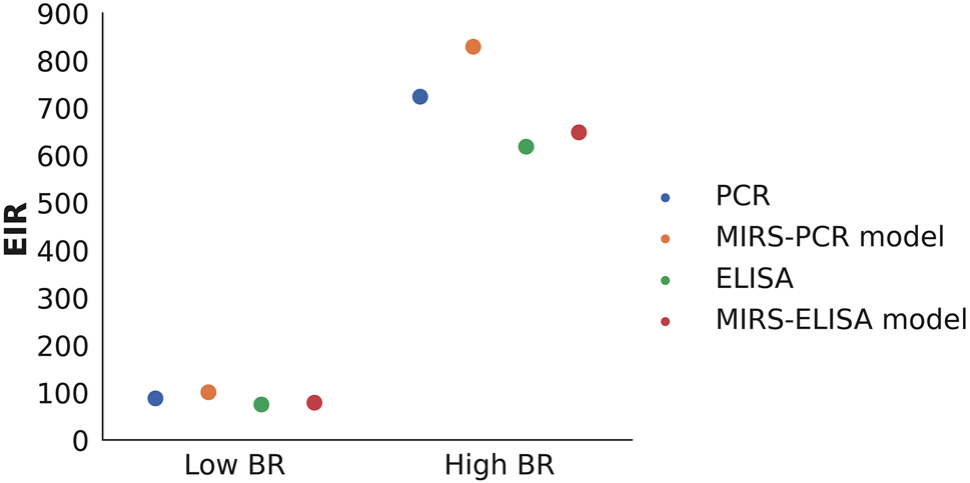
Estimated entomological inoculation rate from MIRS-ML, PCR, and ELISA predictions under hypothetical low and high mosquito biting rates.

Estimation of the EIR was performed using balanced test sets from PCR and ELISA infection datasets used during model testing. Two parameters were used: sporozoite rate and biting rate. The sporozoite rate for PCR and ELISA was calculated as the number of infectious mosquitoes divided by the total number of mosquitoes tested (refer to Table 1). For MIRS prediction, the sporozoite rate was calculated as the number of mosquitoes predicted as infectious (sum of True Positives (TP) and False Positives (FP)) divided by the total number of mosquitoes predicted, as derived from the confusion matrices in Table 1. The low and high biting rates of 0.5 and 4.13, respectively, were sourced from literature as the biting rates for *An. funestus* in the Kilombero valley^29,32^. It was found that, in scenarios with both low and high biting rate, EIR estimates from the MIRS-ML models closely matched the ‘ground truth’ values from PCR and ELISA, showing minimal variation (Fig. 4).

**Table 1:** Displays the balanced, unseen segment of the PCR and ELISA infection datasets alongside their respective machine learning predictions.

	Predicted non-infectious	Predicted Infectious
PCR model prediction on an unseen segment of the PCR infection dataset^1^		
Actual non-infectious	TN = 13 (87%)	FP = 2 (13%)
Actual infectious	FN = 0 (0%)	TP = 14 (100%)
ELISA model prediction on an unseen segment of the ELISA infection dataset^2^		
Actual non-infectious	TN = 20 (91%)	FP = 2 (9%)
Actual infectious	FN = 1 (7%)	TP = 14 (93%)

^1^The total number of samples in the test set: N = 29 (Infectious = 14, Non-infectious = 15)

^2^The total number of samples in the test set: N = 37 (Infectious = 15, Non-infectious = 22)

TN: True Negative, FN: False Negative, FP: False Positive, TP: True Positive.

## Discussion

In the quest for effective malaria control, particularly in regions like sub-Saharan Africa where the burden of this disease is heaviest, the development of rapid, cost-efficient tools for monitoring transmission dynamics is imperative and urgent. Being able to swiftly identify *Plasmodium*-infectious *Anopheles* is particularly critical for understanding the transmission patterns in different localities, estimating the impact of interventions and planning new interventions. Unfortunately, current methodologies, predominantly ELISA and PCR, for detecting *Plasmodium* in *Anopheles* mosquitoes are resource-intensive, necessitating specialized skills and materials often scarce in local settings. This limitation hampers granular, district-level evaluations of malaria risk and the effectiveness of interventions.

Our research presents a novel, economical approach that leverages mid-infrared (MIR) spectroscopy coupled with supervised machine learning algorithms to swiftly identify *Plasmodium*-infectious *Anopheles* mosquitoes. By collecting and analysing the MIR spectral signatures from the heads and thoraces of wild-caught *An. funestus* females in rural Tanzanian villages, and subsequently validating these findings with ELISA or PCR for the presence of *P. falciparum* sporozoite, we established a reliable 'ground truth' for model training. The findings of this study are compelling, demonstrating that MIR spectral analysis can differentiate between infectious and non-infectious mosquitoes with accuracies exceeding 90% in certain cases. Notably, models trained on PCR data showed greater generalizability compared to those based on ELISA data, with mosquito age posing no significant interference. Although tested exclusively on *P. falciparum* and *An. funestus*, this advancement represents a significant step in malaria surveillance. Once calibrated for other major Afro-tropical malaria vectors and malaria transmission systems, it could have the potential to offer a scalable, low-cost solution that could transform data-driven decision-making in disease control programs. Moreover, we view this as an important step towards creating a deployment-ready system but recognize that further development is necessary. Models trained using more diverse data from different settings will improve observed accuracies and enhance the readiness of this approach for broader implementation.

This study contributes to the expanding body of knowledge showcasing the potential of MIRS-ML based approaches for malaria vector surveillance. The use of these methodologies in delineating key entomological parameters such as age, species identification, and blood-feeding patterns of mosquitoes has been well documented^21,22,25^. The outcomes of our study suggest that this technology could serve as a versatile platform, enabling the interpretation of infrared scans to ascertain not only the species and age of mosquitoes, factors critical to their potential as malaria vectors, but also their blood-feeding history on humans or other vertebrates, and their infection status with malaria parasites. Such comprehensive profiling is instrumental in accurately characterizing malaria risk, marking a significant advancement in vector surveillance and malaria control strategies.

In addition to the high classification accuracies of the MIRS-ML approach, the PCR-trained models also achieved generalisability of > 85% in predicting sporozoite infection in wild-caught *An. funestus* mosquitoes even when predicting results of an ELISA dataset. These findings achieve consistent performance with studies utilizing NIRS frequencies in the laboratory, which reported a > 90% classification accuracy in detecting *P. falciparum* sporozoite infection in *An. gambiae* mosquitoes^15^, and 77% accuracy in detecting *P. berghei* sporozoite infection in *An. stephensi*^16^. While earlier models trained on NIRS failed to identify mosquitoes infected with wild-strain parasites from asymptomatic malaria carriers, possibly due to limitations in the training dataset or detection capabilities of the system^33^, models trained on MIRS, which provide clearer peaks with richer biochemical information appear to perform better^18,19^. This enhancement enabled our models to effectively identify infections in mosquitoes, a capability not fully realized with NIRS models in previous studies.

MIRS captures the biochemical composition of mosquito, which may consistently differ, in this case, with the infection status such as presence or absence of the parasite. The presence of parasite-specific proteins, such as circumsporozoite (CS) protein and the thrombospondin-related adhesive protein (TRAP), may contribute to the main spectral difference between infectious and non-infectious *An. funestus*^34^. Furthermore, since mosquitoes elicit immune responses to the parasites, this could consequently affect the biochemical characteristics of the infectious or non-infectious mosquitoes^35^. Additionally, higher levels of energy resource storage, such as glucose and lipid accumulation in the non-infectious mosquitoes^36,37^, might yield distinct spectra signals between infectious and non-infectious *An. funestus*. This aligns with our observation where the majority of spectral features influencing machine learning prediction primarily originated from the O–H, C-H, and N–H bonds, as well as the fingerprint region of the spectrum (1500 cm^−1^ to 520 cm^−1^), suggesting the presence of carbohydrates, protein, and lipids related to the parasite^20^. However, it is important to note that we are not focusing on individual spectra features; rather, we are using ML models to integrate a set of spectral features from different biochemical group regions to enable these classifications. While it may not be essential to identify specific features, we believe that additional studies should be conducted to better understand the biochemical signals underlaying our algorithmic classifications.

The biological prerequisite that mosquitoes must exceed a certain age threshold (e.g. over 9 days) to become vectors for malaria transmission, due to the requisite extrinsic incubation period for the parasite^38^, introduces potential age-related biases in detection efficacy. In this context, mosquito age could be considered a confounding factor influencing prediction accuracy. However, despite the theoretical possibility of age influencing the accuracy of predictions, our analysis demonstrated that the machine learning models adeptly identified signals indicative of infection across all age brackets, including older mosquitoes beyond 14 days, thus negating age as a significant confounding variable in our study.

Moreover, the ML model trained with the PCR infection dataset demonstrated an ability to generalise its prediction to samples screened by ELISA. In contrast, the model trained with the ELISA infection dataset had some limitation in predicting samples screened by PCR. We further observed similarities in the fingerprint region where both ELISA and PCR models detected signals, demonstrating agreement in parasite detection between the two models (Fig. 3). However, a distinct difference was noted on the signals detected by both ELISA and PCR models, particularly in the frequencies ranging from 3500 to 3000 cm^−1^. Moreover, it is still not clear why ML models are picking up different signals from this region. Additionally, the generalisability of the ML model trained with PCR infection dataset can be attributed to the sensitivity of PCR in detecting even low sporozoite numbers in mosquitoes^39^. Leveraging the sensitivity of PCR can enhance the performance of MIRS-ML models. However, a study by Hendershot et al*.,* observed infection in mosquitoes at 0.5–1 day post-infection, indicating that false positive results can occur because PCR can report positives even when sporogonic development has not started^39^. This situation arises when an infectious blood meal has not full migrated to the mosquito abdomen, and the presence of gametocytes in the mosquito head and thorax is more likely to contribute to positive results.

The primary focus of this investigation was to showcase the field application of the MIRS-ML technique for detecting sporozoites in malaria vectors, not to directly compare it with PCR or ELISA methods, which were instead used solely to provide reference labels for ML model training. Moreover, this study represents only the first demonstration of field applications of the MIRS-ML technique for sporozoite detection in malaria vectors, underscoring the need for further validation before its integration into surveillance or national malaria control efforts. Our analysis was also confined to *An. funestus* mosquitoes, chosen for their relatively high sporozoite rates in the region, highlighting the necessity to broaden future models to include more vector species. We recognize that expanding the MIRS-ML approach to all important mosquito species may necessitate compiling a comprehensive dataset of mosquito infection spectra, a task that presents logistical challenges in field settings, especially where natural infection prevalences are very low. A promising solution is to employ transfer learning, integrating laboratory-generated data with field-collected samples to enhance model robustness^24,25^. This method involves refining a model initially trained on laboratory data with new field data, facilitating the development of an effective tool for field infection prediction. Additionally, in low transmission setting where sporozoite infection rates are low, ELISA and PCR can be used for mosquito pool testing, reducing operational costs compared to individual mosquito tests. However, the feasibility of using MIRS-ML for mosquito pool testing remains unknown, prompting our investigation in the next steps. Additionally, in this study, MIRS-ML was not evaluated for identifying the *Plasmodium* species. Future studies should address this aspect to enhance the utility in regions where more than once species of *Plasmodium* is prevalent.

MIRS-ML proves cost-effective as it eliminates the need for repeated reagent costs in mosquito sample tests, with the only incurred expense being the initial £ 25,000/ = for purchasing the FT-IR spectrometer. Capable of processing approximately 60 mosquitoes per hour, the portable bench-top design of the FT-IR spectrometer measures 22 × 33 × 26 cm, requiring connection to an AC power supply. Currently, we are developing an online system to serve as a centralized platform for predicting various entomological and parasitological indicators of malaria. This online system aims to facilitate the scaling up of MIRS-ML, enabling end-users from different locations to upload unknown mosquito spectra for predictions related to infection status, species, age, or resistance status.

In conclusion, here we demonstrate the first application of mid-infrared spectroscopy combined with machine learning (MIRS-ML) for the rapid and accurate detection of *P. falciparum* in field collected mosquitoes. By analysing 7178 female *An. funestus* specimens collected from rural Tanzania, we achieved detection accuracies of 92% and 93% against ELISA and PCR benchmarks, respectively. Moreover, MIRS-ML can guide programmatic decisions on vector control, as the EIR estimates, derived from MIRS-ML models, closely align with those obtained from PCR and ELISA methods across low and high biting rate scenarios, demonstrating consistency and reliability in malaria infection prediction. This method, capable of processing approximately 60–100 mosquitoes per hour with minimal costs, presents a significant advancement in malaria surveillance, particularly in sub-Saharan Africa where the disease has a profound impact. The utility of MIRS-ML extends beyond sporozoite detection, offering insights into critical entomological indicators such as mosquito age, blood-feeding patterns, and species identification, thereby positioning MIRS-ML as a versatile tool in malaria risk assessment and evaluation of vector control interventions.

## Methods

### Mosquito collection and processing

**Figure 5:**
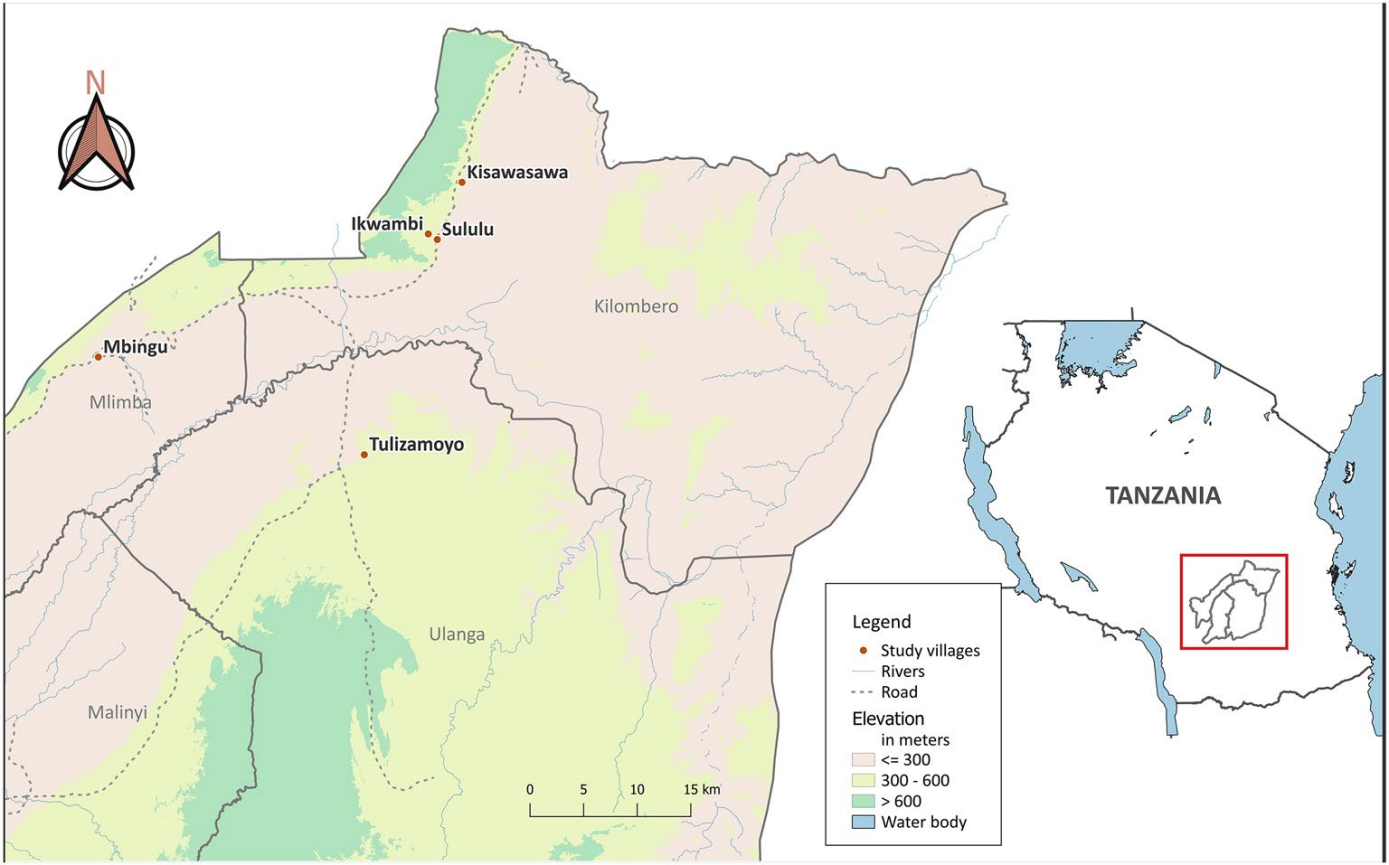
Map of the five villages where mosquitoes were collected.

Mosquitoes were collected from five villages in two rural districts in South-eastern Tanzania, Kilombero and Ulanga: Kisawasawa (7.8941°S, 36.8748°E), Mbingu (8.1952°S, 36.2587°E), Ikwambi (7.9824°S, 36.8216°E), Sululu (7.9973°S, 36.8317°E) and Tulizamoyo (8.3544°S, 36.7054°E) (Fig. 5). These villages experience annual rainfall of 1200–1800 mm, with mean daily temperatures of 20–32 °C^40^, and were selected because of the high densities of the malaria vector, *An. funestus.* The vector species was chosen for this study because it is the primary contributor to malaria transmission in the area and typically exhibits a higher prevalence of *Plasmodium* sporozoites compared to other local vector species such as *Anopheles arabiensis*^29,30^. Mosquitoes were collected both indoors using CDC light traps and Prokopack aspirators^41,42^, and outdoors, in outdoor kitchens and animal sheds using resting buckets^43^. The collected *Anopheles* mosquitoes were sorted by taxa based on their morphological features^44^. All *An. funestus* group mosquitoes were immediately killed with chloroform and then stored individually in 1.5 mL microcentrifuge tubes containing silica gel as desiccant and preservative. The *An. funestus* samples were transferred to the VectorSphere laboratory at the Ifakara Health Institute and stored dry for at least five days for further investigation.

### Mid-infrared spectroscopy

We used a Bruker ALPHA II Fourier-Transform Infrared (FT-IR) spectrometer with attenuated total reflectance (ATR) to measure the infrared spectrum of the dried mosquito samples. Prior to scanning, the head and thorax of each mosquito was carefully separated from the abdomen, ensuring that only the head and thorax regions were scanned. The mosquito heads and thoraces were placed on the infrared optical window, and pressure was applied to ensure maximum direct contact between the sample and the diamond crystal. The spectral signal was obtained at frequencies between 4000 and 400 cm^−1^, with a resolution of 2 cm^−1^. Each spectrum was an average of 32 scans of a single mosquito sample, with band intensity recorded as an absorbance. Following scanning, the remaining of the mosquito head and thorax (carcasses) were individually packed in 1.5 mL tubes for subsequent molecular analysis. The recorded spectra were pre-processed by compensating for carbon dioxide interference bands and water vapor absorption bands as previously described^22^. Additionally, spectra with no intensity (i.e. flat spectra) and low intensity (i.e. < 0.11 absorbance units) were removed before machine learning steps^22^.

### Detection of *Plasmodium sporozoites* using PCR and ELISA

To obtain reference labels of *P. falciparum* sporozoite infections in the mosquito head and thorax carcasses, we used real-time PCR targeting var gene acidic terminal sequences (*varATS*) of the parasites^9,10^ and Enzyme-linked immunosorbent assays (ELISA) assays for detecting circumsporozoite protein (CSP)^8^. Each carcass underwent individual analysis, a method previously demonstrated for detecting mosquito blood meal remaining^45^. In total, 7178 *An. funestus* carcasses were examined across two rounds using the following methods:

Initially, 4281 samples were screened using ELISA^8^, each time ensuring that the lysates of all the positive samples were boiled for 10 min at 100˚C to eliminate false positives usually associated with heat labile non-*Plasmodium* protozoans, and retested^12^.

Next, 2897 samples underwent multiplex real-time PCR for sporozoite infection detection. This involved DNA extraction of mosquito carcasses using the DNAzol® reagent^46^, DNA was eluted in 50 µL of Tris–Acetate-EDTA (TAE) buffer. Subsequent to this, Real-Time PCR was conducted targeting the Pan*-Plasmodium* 18S rRNA and *P. falciparum* specific *varATS* sequences, along with a 28S rRNA mosquito sequence as a reference gene/internal control, enhancing specificity and sensitivity for *P. falciparum* and non-*falciparum* species.

The PCR reaction used a 10 µl mix including Luna Universal Probe qPCR Master Mix (New England Biolabs, USA), a primer mix, water, and template DNA. The thermal cycle parameters involved an initial polymerase activation at 95 °C for 1 min, DNA denaturation at 95 °C for 15 s for 45 cycles, and annealing/elongation at 57 °C for 45 s for 45 cycles^10,47^. Samples exhibiting a sigmoid curve that reached the cycle threshold (Ct) value at ≤ 35 cycles were classified as positive, while those reaching > 35 cycles were classified as negative. The assays were run in duplicates, and each run included a non-template control and *P. falciparum* NF54 DNA as positive control. The real-time PCR measurements were analysed using CFX96 Real-Time PCR system (Bio-Rad Laboratories, USA).

### Data analysis

The PCR and ELISA data were separately used as references to evaluate performance of the infrared spectroscopy and machine leaning models for accurately identifying individual mosquitoes infected with *P. falciparum* sporozoites in their salivary glands. Since only a small proportion of the mosquitoes were found infectious (see results section), it was necessary to first obtain similar numbers of randomly selected non-infectious mosquitoes as controls, to avoid skewed model performance. The non-infectious samples were therefore under-sampled by randomly selecting individual specimen based on their smallest average Euclidian distances to the 3 farthest positive samples^48,49^. This process was repeated 50 times and bootstrapped to cover as many negative samples as possible.

To ensure consistency and uniformity, the spectra data were standardized using the *StandardScaler* algorithm^50^. Supervised machine learning techniques, including K-nearest neighbours (KNN), logistic regression (LR), support vector machine (SVM), gradient boosting (XGB), random forest (RF), and multilayer perception (MLP), were then compared for predicting the ELISA and PCR results. The model with the highest accuracy was optimized further by adjusting its hyperparameters using randomized search cross-validation, and its final estimator was evaluated using *K-fold* cross-validation (k = 5). The analysis was performed using Python 3.8 with the *Scikit-learn* library^50^. The machine learning models were trained using ELISA, PCR, or combined ELISA + PCR training datasets and tested on all three corresponding test sets (Table 2). Training was done with up to 90% of the known positive and negative samples, each time leaving out at least 10% for model validation (Table 2). Additional validation of the models included using samples tested by either of the two methods, and incorporating lab-reared, non-infectious mosquitoes confirmed to be at least 14 days old. This was to guarantee that the models accurately classified infection status rather than mosquito age, as age can confound results and only mosquitoes older than 9 days are capable of transmitting malaria^38^.

**Table 2:** Training and test datasets used in the different models.

Model	Training data	Test data
ELISA	ELISA dataset (90%)	1. ELISA dataset (10%) 2. ELISA dataset (10%) modified (14-day old lab-reared *An. funestus* mixed into negative class) 3. PCR dataset
PCR	PCR dataset (90%)	1. PCR dataset (10%) 2. PCR dataset (10%) modified (14-day old lab-reared *An. funestus* mixed into negative class) 3. ELISA dataset
Combined	PCR & ELISA dataset combined (90%)	1. PCR & ELISA dataset combined (10%)

### Ethics approval and consent to participate

Ethical approval for this study was obtained from the Institutional Review Board at Ifakara Health Institute (Ref. IHI/IRB/No: 41-2020), and the Medical Research Coordinating Committee (MRCC) at the National Institute of Medical Research (NIMR) (Ref: NIMR/HQ/R.8a/Vol. IX/3557). Since this study focused primarily on malaria mosquitoes, it did not involve human participants or animals.

## Data Availability

The mid-infrared spectral datasets generated and analysed, code for analyses during the current study are deposited and available at: https://github.com/MwangaEP/Sporozoite-detection-funestus.

## Abbreviations

MIRS: Mid-infrared spectroscopy
NIRS: Near-infrared spectroscopy
PCR: Polymerase chain reaction
ELISA: Enzyme-linked immunosorbent assay
FT-IR: Fourier-transform Infrared spectrometer
MIRS-ML: Mid-infrared spectroscopy and machine learning
